# Prevalence of depression and its associated factors in bronchiectasis: findings from KMBARC registry

**DOI:** 10.1186/s12890-021-01675-4

**Published:** 2021-09-27

**Authors:** Ji-Ho Lee, Won-Yeon Lee, Suk Joong Yong, Woo Jin Kim, Sooim Sin, Chang Youl Lee, Youlim Kim, Ji Ye Jung, Sang-Ha Kim, the KMBARC, Yeon-Mok Oh, Hyun Lee, Hayoung Choi, Yun Su Sim, Kwang Ha Yoo, Seung Jun Lee, Tae-Hyung Kim, Bumhee Yang, Ina Jeong, Soo-Jung Um, Deog Kyeom Kim, Ji-Hyun Lee, Byoung Soo Kwon, Young-Jae Cho, Chang-Hoon Lee, Chin Kook Rhee, Sang Haak Lee, Ju-Ok Na, An-Soo Jang, Changhwan Kim, Hyun Kuk Kim, Hye Yun Park, Jae Seung Lee, Sei Won Lee, Seung Won Ra, Sung-Yoon Kang, Yee Hyung Kim, Yong Bum Park, So-Young Park, Junghyun Kim, Young-Soon Yoon, Yun Jeong Jeong, Jung-Kyu Lee, Ki Uk Kim, Hyun-Kyung Lee, Eun Kyung Kim, Se Hee Lee, Jae Sung Choi, Hyung Koo Kang, Yong-Soo Kwon, Jae Ha Lee

**Affiliations:** 1 Department of Internal Medicine, Yonsei University Wonju College of Medicine, 20 Ilsan-ro, Wonju, 26426 Korea; 2 Department of Internal Medicine and Environmental Health Center, Kangwon National University, Chuncheon, Korea; 3 Department of Internal Medicine, Kangwon National University Hospital, Gangwon-do, Chuncheon-si, Korea; 4 Department of Internal Medicine, Hallym University Chuncheon Sacred Heart Hospital, Chuncheon, Korea; 5 Division of Pulmonary and Critical Care Medicine, Department of Internal Medicine, Severance Hospital, Yonsei University College of Medicine, Seoul, Korea

**Keywords:** Bronchiectasis, Depression, Dyspnea, mMRC, Exacerbation

## Abstract

**Background:**

With the emergence of bronchiectasis as a common respiratory disease, epidemiological data have accumulated. However, the prevalence and impact of psychological comorbidities were not sufficiently evaluated. The present study examined the prevalence of depression and its associated factors in patients with bronchiectasis.

**Methods:**

This study involved a multicenter cohort of bronchiectasis patients recruited from 33 pulmonary specialist hospitals. The baseline characteristics and bronchiectasis-related factors at enrollment were analyzed. Depressive symptoms were assessed using the Patient Health Questionnaire (PHQ-9).

**Results:**

Of the 810 patients enrolled in the study, 168 (20.7%) patients had relevant depression (PHQ-9 score ≥ 10), and only 20 (11.9%) patients had a diagnosis of depression. Significant differences were noted in the depressive symptoms with disease severity, which was assessed using the Bronchiectasis Severity Index and E-FACED (all *p* < 0.001). Depressive symptoms inversely correlated with quality-of-life (r = − 0.704, *p* < 0.001) and positively correlated with fatigue severity score (r = 0.712, *p* < 0.001). Multivariate analysis showed that depression was significantly associated with the modified Medical Research Council dyspnea scale ≥ 2 (OR 2.960, 95% CI 1.907–4.588, *p* =  < 0.001) and high number of exacerbations (≥ 3) in the previous year (OR 1.596, 95% CI 1.012–2.482, *p* = 0.041).

**Conclusions:**

Depression is common, but its association with bronchiectasis was underrecognized. It negatively affected quality-of-life and presented with fatigue symptoms. Among the bronchiectasis-related factors, dyspnea and exacerbation were closely associated with depression. Therefore, active screening for depression is necessary to optimize the treatment of bronchiectasis.

***Trial registration*:**

The study was registered at Clinical Research Information Service (CRiS), Republic of Korea (KCT0003088). The date of registration was June 19th, 2018.

## Background

Bronchiectasis is characterized by permanent dilation of the bronchus and recurrent respiratory symptoms [1]. It is the third most common chronic airway disease following asthma and chronic obstructive pulmonary disease (COPD) [2]. Bronchiectasis is a common respiratory disease, and epidemiological data have accumulated. The prevalence of bronchiectasis is 139 per 100,000 people in the United States [3]. The prevalence of bronchiectasis in the United Kingdom is 566 per 100,000 for women and 485 per 100,000 for men [4]. A study of Asian countries found that it was 1.2% in China and 464 per 100,000 people in South Korea [5, 6].

Gastroesophageal reflux disease, cardiovascular disease, osteoporosis, asthma, and COPD are common comorbidities in bronchiectasis [7]. Psychological comorbidities are more common in patients with chronic airway disease than the general population [8]. The coexistence of psychological comorbidities negatively affects clinical outcomes, such as worsening of symptoms, frequent exacerbations, poor adherence to treatment, and increasing mortality [9]. Depression also commonly coexists with bronchiectasis [10]. However, the prevalence of depression, its impacts and associated factors were not sufficiently identified compared to other chronic airway diseases.

Most studies on depression in bronchiectasis were performed at a single medical center [11]. As a result of insufficient subjects, their research results are difficult to generalize. The present study determined the prevalence of depression and examined the relationship between depression and quality-of-life and the clinical factors associated with bronchiectasis in a larger sample of bronchiectasis patients using a validated questionnaire.

## Methods

### Study design

The data source of this study was the Korean Multicenter Bronchiectasis Audit and Research Collaboration (KMBARC) registry. The KMBARC was an observational cohort study that examined the clinical characteristics, disease burden, etiology, risk factors for exacerbation, and prognosis in Korean patients with bronchiectasis. Patients were included if they were aged ≥ 18 years and had bronchiectasis confirmed by computed tomography in one or more lobes. Patients with cystic fibrosis and secondary traction bronchiectasis were excluded. All baseline data at the time of enrollment were collected when patients were stable for 4 weeks. Data on age, body mass index (BMI), pulmonary function tests, modified Medical Research Council (mMRC) dyspnea scale score, exacerbation, etiology determined by physicians, microbiological results, and CT findings were collected at baseline. Further information on the protocol was recently published [12]. The patients were enrolled between August 2018 and November 2020 from 33 pulmonary specialist hospitals. Baseline data were extracted during the enrollment of the cohort, and analyses were performed.

The Institutional Review Board of Wonju Severance Christian Hospital (CR318139) and the institutional review boards of all participating institutions approved the study, and it adhered to the principles of the Declaration of Helsinki. Written informed consent was obtained from all patients at the time of study enrollment.

### Definition of study variables

Exacerbation of bronchiectasis was defined as an aggravation of three or more symptoms, including cough, sputum volume and/or consistency, sputum purulence, dyspnea and/or exercise intolerance, fatigue and/or malaise, and hemoptysis, for at least 48 h that necessitated a change in treatment [13]. Exacerbations were assessed using information obtained via history taking and reviews of antibiotic prescription records and medical charts. The number of exacerbations and rate of hospital admissions within the previous 12 months before enrollment in the study were recorded.

The severity of bronchiectasis was evaluated using validated scoring systems, such as the Bronchiectasis Severity Index (BSI), FACED, and E-FACED. BSI takes into account age, BMI, predicted value of forced expiratory volume in the first second (FEV_1_%), number of admissions and exacerbations, mMRC dyspnea scale, *Pseudomonas aeruginosa* (PA) colonization, and bronchiectasis involving 3 or more lobes or the presence of cystic bronchiectasis. According to the BSI, the severity of bronchiectasis was categorized into mild (0–4), moderate (5–8), and severe (≥ 9) [14]. FACED stands for FEV_1_% (F), age (A), colonization with PA (C), extension of bronchiectasis (E), and dyspnea score for mMRC (D). FACED was categorized into mild (0–2), moderate (3–4), and severe (5–7) [15]. Exacerbation was added to the FACED in E-FACED, and it was classified into mild (0–3), moderate (4–6), and severe (7–9) [16].

The first coronavirus disease 2019 (COVID-19) occurred on January 20, 2020, in our country. The COVID-19 pandemic developed from March 2020. Therefore, all participants were classified into pre-pandemic (until February 2020) and pandemic (from March 2020) groups according to the enrollment date to determine whether COVID-19 affected the prevalence of depression.

### Patient-reported outcomes

This study used three patient health-related questionnaires. The Bronchiectasis Health Questionnaire (BHQ) was developed to measure bronchiectasis-specific quality-of-life and health status. The BHQ is comprised of 10 items, with higher scores indicating a better quality-of-life [17]. A Korean version of the BHQ that was validated in Korean patients with bronchiectasis was used [18]. Fatigue was assessed using the Korean version of the Fatigue Severity Score (FSS) [19]. The FSS is comprised of 9 items. Higher FSS indicates more severe fatigue symptoms.

The Patient Health Questionnaire 9 (PHQ-9) was used to assess depressive symptoms. The PHQ-9 contains 9 items. Each item is evaluated on a 4-point scale: 0, “not at all”; 1, “several days”; 2, “more than half of the days”; and 3, “nearly every day.” Higher scores on the PHQ-9 indicate more severe depressive symptoms. A Korean version of the PHQ-9 was validated for screening major depression [20]. Patients with a PHQ-9 score of 10 or greater were considered to have depression. All three questionnaires were administered in the outpatient department after the patients were stable for at least 4 weeks.

### Statistical analysis

Continuous variables are presented as the means ± standard deviations, and categorical variables are presented as the number of patients and percentages. The baseline clinical characteristics of patients with and without depression were compared using an independent *t*-test for continuous variables, and a chi-squared test was used for categorical variables. PHQ-9 scores for the degrees of severity assessed by the BSI, FACED, and E-FACED were compared using a one-way analysis of variance with post hoc testing using the Bonferroni test. Correlations between the continuous variables and PHQ-9 scores were assessed using the Pearson correlation coefficient (r). Binary logistic regression was used to identify the factors associated with depression. Statistical analyses were performed and the figures were prepared using R software (ver. 4.0.3; R Development Core Team, Vienna, Austria). Statistical significance was set at *p* < 0.05.

## Results

### Baseline characteristics

Among 848 patients in a registry, patients lacking demographics or missing data for critical values for analysis were excluded. A total of 810 patients with bronchiectasis were ultimately included for this study (Table 1). The mean age was 64.3 ± 9.3 years, and 452 (55.8%) of the participants were female. There were 168 (20.7%) patients with depression (PHQ-9 ≥ 10), and only 20 (11.9%) patients had a diagnosis of depression. The number of recruited patients was 665 in the pre-pandemic group and 145 in the COVID-19 pandemic group. The prevalence of depression was 21.7% (144/665) pre-pandemic and 16.6% (24/145) pandemic. A significant difference was not found between these groups (*p* = 0.208). The baseline characteristics of patients with and without depression were compared. Patients with depression had a lower BMI than patients without depression (22.5 ± 3.9 versus 23.3 ± 6.9, *p* = 0.049). Lung function was significantly lower in patients with depression without depression. FEV_1_ was 60.4 ± 20.8% and 65.8 ± 20.8% for patients with and without depression, respectively (*p* = 0.005). FVC was 68.4 ± 17.1% and 74.1 ± 17.5% for patients with and without depression, respectively (*p* < 0.001). The number of exacerbations and rates of hospital admission within the previous 12 months were significantly higher for patients with depression than patients without depression (number of exacerbations: 2.0 ± 3.7 versus 1.2 ± 1.9, *p* < 0.001; admission rate: 25.0% versus 15.4%, *p* = 0.005). The number of patients treated with long-term oxygen therapy (LTOT) or noninvasive ventilation (NIV) were 11 (6.5%) and 8 (1.2%) patients with and without depression, respectively (*p* < 0.001). The number of patients admitted to the intensive care unit or treated with mechanical ventilation for the management of bronchiectasis exacerbations were 3 (1.8%) for patients with depression and 4 (0.6%) for patients without depression (*p* = 0.326).

**Table 1:** Baseline characteristics of study subjects and comparison between patients with and without depression

Variable	Nondepressed (PHQ-9 < 10) (n = 642)	Depressed (PHQ-9 ≥ 10) (n = 168)	*p* value
Age (years)	64.6 ± 9.1	63.3 ± 10.2	0.140
Sex (female)	355 (55.3)	97 (57.7)	0.631
Bronchiectasis duration (years)	3.2 ± 1.6	3.3 ± 1.7	0.432
Ever smoker	216 (33.7)	56 (33.3)	1.000
BMI	23.3 ± 6.9	22.5 ± 3.9	0.049
FEV_1_ (%)	65.8 ± 20.8	60.4 ± 20.8	0.005
FEV_1_ (L)	1.8 ± 0.6	1.6 ± 0.6	0.002
FVC (%)	74.1 ± 17.5	68.4 ± 17.1	0.001
FVC (L)	2.7 ± 0.8	2.4 ± 0.8	< 0.001
Asthma or COPD	294 (45.8)	80 (47.6)	0.737
Number of exacerbations in the previous year	1.2 ± 1.9	2.0 ± 3.7	< 0.001
Hospitalization	99 (15.4)	42 (25.0)	0.005
Tuberculosis history	219 (35.8)	51 (31.7)	0.372
NTM history	65 (10.64)	14 (8.70)	0.564
Depression history	15 (2.3)	20 (11.9)	< 0.001
LTOT or NIV	8 (1.2)	11 (6.5)	< 0.001
ICU or MV	4 (0.6)	3 (1.8)	0.326
*Etiology*			
Post-infectious	249 (43.7)	60 (38.5)	0.281
Idiopathic	229 (40.2)	66 (42.3)	0.698
PA colonization	46 (7.3)	16 (9.6)	0.412
Sputum volume (≥ 30 cc/day)	253 (39.4)	64 (38.1)	0.825
Radiological extent (≥ 3 lobes)	342(53.3)	96 (57.1)	0.418

Values are presented as the means ± standard deviation or number (%)

BMI, body mass index; FEV_1_, forced expiratory volume in 1 s; FVC, forced vital capacity; NTM, nontuberculosis mycobacterium; LTOT, long term oxygen therapy; NIV, noninvasive ventilation; ICU, intensive care unit; MV, mechanical ventilation; PA, *Pseudomonas aeruginosa*

The severity of bronchiectasis and patient-reported outcomes were compared between patients with and without depression (Table 2). The mean BSI and E-FACED scores were significantly higher for patients with depression than patients without depression (BSI: 8.0 ± 4.7 versus 6.6 ± 3.4, *p* = 0.001; E-FACED: 2.8 ± 2.3 versus 2.3 ± 1.8, *p* = 0.014). However, the severity estimated by FACED did not differ (*p* = 0.425). The quality-of-life score assessed using the BHQ was 41.4 ± 9.5 for patients with depression, which was lower than 54.8 ± 7.4 for patients without depression (*p* < 0.001). The fatigue score estimated by the FSS was 39.7 ± 13.6 for patients with depression, which was higher than 20.0 ± 11.1 for patients without depression (*p* < 0.001).

**Table 2:** Comparison of severity index and patient-reported outcomes between patients with and without depression

Variable	Nondepressed	Depressed	*p* value
BSI	6.6 ± 3.4	8.0 ± 4.7	0.001
FACED	2.3 ± 1.6	2.4 ± 1.7	0.425
E-FACED	2.3 ± 1.8	2.8 ± 2.3	0.014
BHQ	54.8 ± 7.4	41.4 ± 9.5	< 0.001
FSS	20.0 ± 11.1	39.7 ± 13.6	< 0.001

Values are presented as the means ± standard deviation

BSI, bronchiectasis severity index; BHQ, Bronchiectasis Health Questionnaire; FSS, Fatigue Severity Score

### Depressive symptoms across the severity of bronchiectasis

**Fig. 1:**
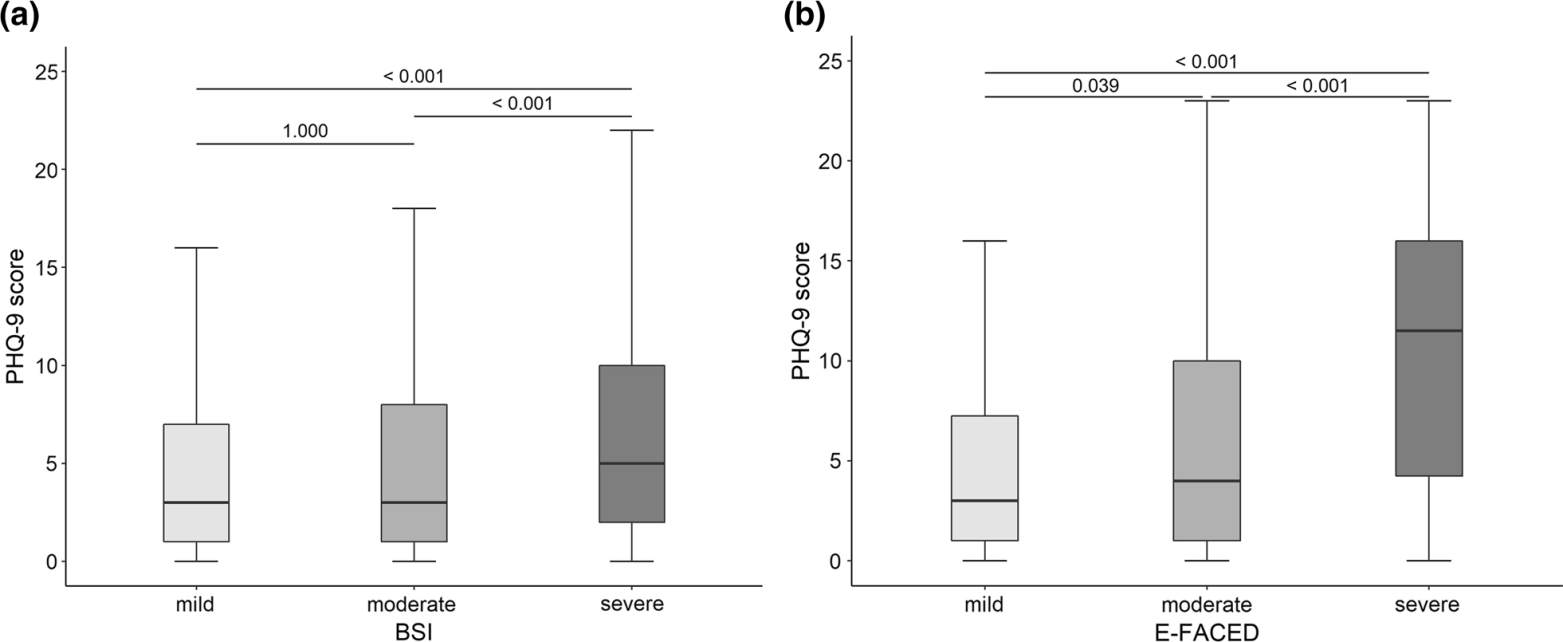
Comparison of PHQ-9 scores according to the severity classified by BSI (**a**) and E-FACED (**b**). The number at the top of the figure indicates the *p* value from the Bonferroni post hoc test. *PHQ-9* Patient Health Questionnaire 9, *BSI* bronchiectasis severity index

The PHQ-9 scores were compared according to the grade of severity. The PHQ-9 scores were 4.8 ± 5.6, 4.8 ± 5.0, and 6.9 ± 6.7 for the mild, moderate, and severe groups based on the BSI, respectively (*p* < 0.001). The post hoc analysis showed that the PHQ-9 scores for the mild versus severe and moderate versus severe groups were significantly different (all *p* < 0.001) (Fig. 1a). PHQ-9 scores for the E-FACED were 4.9 ± 5.4 for the mild group, 6.2 ± 6.4 for the moderate group, and 11.1 ± 6.9 for the severe group (*p* < 0.001). The post hoc analysis showed that the PHQ-9 scores for the mild versus moderate, mild versus severe, and moderate versus severe groups were significantly different (*p* = 0.039, *p* < 0.001, and *p* < 0.001, respectively) (Fig. 1b).

### Relevant factors underlying depression in bronchiectasis

**Fig. 2:**
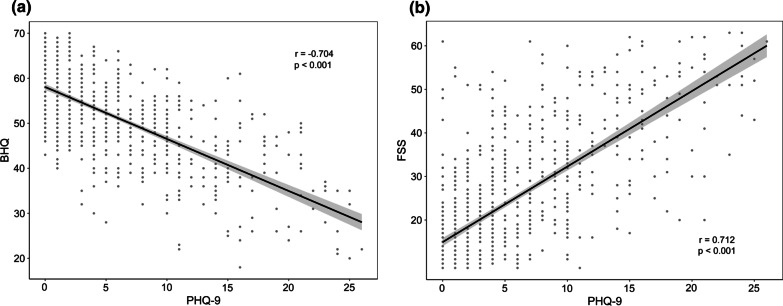
Results of correlation analysis between the PHQ-9 and BHQ (**a**) and FSS (**b**). PHQ-9 scores inversely correlated with the BHQ and positively correlated with the FSS. *PHQ-9* Patient Health Questionnaire 9, *BHQ* Bronchiectasis Health Questionnaire, *FSS* Fatigue Severity Score

PHQ-9 scores inversely correlated with BHQ (r = − 0.704, *p* < 0.001) and positively correlated with FSS (r = 0.712, *p* < 0.001) (Fig. 2a, b), and these scores showed a weak positive correlation with BSI (r = 0.214, *p* < 0.001) and E-FACED scores (r = 0.145, *p* < 0.001) and a weak negative correlation with FEV_1_% (r = − 0.134, *p* < 0.001).

The univariate analysis showed that FEV_1_% was negatively associated with depression (odds ratio [OR] = 0.985, 95% confidence interval [CI]: 0.976–0.994, *p* = 0.001). The mMRC dyspnea scale was associated with an elevated risk of depression (OR = 2.031, 95% CI 1.659–2.500, *p* < 0.001). The number of exacerbations and presence of hospital admission were significantly related to depression (exacerbations: OR = 1.140, 95% CI 1.064–1.228, *p* < 0.001; admission: OR = 1.828, 95% CI 1.205–2.740, *p* = 0.004). LTOT or NIV was significantly associated with depression (OR = 5.553, 95% CI 2.211–14.562, *p* < 0.001) (Table 3).

**Table 3:** Factors associated with depression in logistic regression analysis

Factors	Univariate analysis		Multivariate analysis	
	OR (95% CI)	*p* value	OR (95% CI)	*p* value
Age	0.986 (0.968–1.004)	0.114	0.980 (0.960–1.000)	0.045
Sex (female)	1.105 (0.785–1.561)	0.570	1.019 (0.692–1.506)	0.923
BMI	0.952 (0.904–1.002)	0.062	0.971 (0.919–1.024)	0.280
FEV1 (%)	0.985 (0.976–0.994)	0.001	1.001 (0.991–1.012)	0.803
FVC (%)	0.983 (0.972–0.992)	< 0.001	–	–
mMRC	2.031 (1.659–2.500)	< 0.001	2.137 (1.671–2.752)	< 0.001
Number of exacerbations in the previous year	1.140 (1.064–1.228)	< 0.001	1.085 (0.997–1.178)	0.052
Hospitalization	1.828 (1.205–2.740)	0.004	–	–
LTOT or NIV	5.553 (2.211–14.562)	< 0.001	1.899 (0.642–5.673)	0.243

BMI, body mass index; FEV_1_, forced expiratory volume in 1 s; FVC, forced vital capacity; mMRC, Modified Medical Research Council; LTOT, long term oxygen therapy; NIV, noninvasive ventilation; OR, odds ratio; CI, confidence interval

**Fig. 3:**
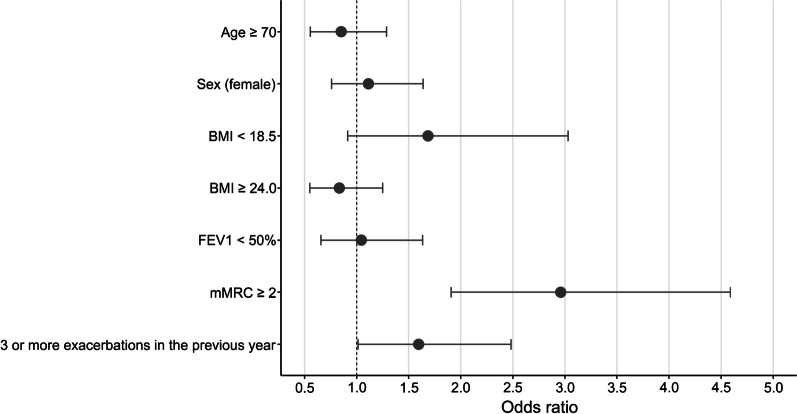
Odds ratios predictive of depression using multivariate analysis

The multivariate analysis showed that the mMRC dyspnea scale was significantly associated with depression (OR = 2.137, 95% CI 1.671–2.752, *p* < 0.001). The number of exacerbations in the previous year and LTOT or NIV were not significantly associated with depression (number of exacerbations: OR = 1.085, 95% CI 0.997–1.178, *p* = 0.052; LTOT or NIV: OR = 1.899, 95% CI 0.642–5.673, *p* = 0.243). A multivariate analysis of the categorical variables was also performed, and mMRC ≥ 2 and a high number of exacerbations (≥ 3) were significantly related to depression (mMRC: OR = 2.960, 95% CI 1.907–4.588, *p* < 0.001; exacerbation: OR = 1.596, 95% CI 1.012–2.482, *p* = 0.041) (Fig. 3). LTOT or NIV was not significantly associated with depression (OR = 2.641, 95% CI 0.961–7.500, *p* = 0.060). Hospitalization was excluded from the multivariate analysis because exacerbation and admission substantially overlapped and interfered with each other.

## Discussion

This multicenter study found a 20.7% prevalence of depression in patients with bronchiectasis. The depressive symptoms were aggravated as bronchiectasis worsened and strongly correlated with quality-of-life and fatigue symptoms. Dyspnea and frequent exacerbations were identified as clinical factors associated with depression.

The prevalence of depression using the Hospital Anxiety and Depression Scale (HADS) was 22.8% in 93 bronchiectasis patients in a cross-sectional study [11]. Other studies using HADS reported a prevalence of 21.1% in 133 patients [21] and 30.1% in 163 patients [22]. A study of depression using the Beck Depression Inventory (BDI) found a prevalence of 33.8% in 74 patients [23]. Previous studies used the HADS or BDI as a screening tool, but our study used the PHQ-9 to assess depressive symptoms. The BDI is comprised of 21 items, and it takes time to complete. The HADS has a low diagnostic accuracy for identifying depression in COPD [24]. The PHQ-9 is comprised of 9 questions that correspond to the diagnostic criteria of depressive disorder and it was an accurate screening tool for depression in a recent meta-analysis with a sensitivity of 88% and specificity of 85% [25]. Because this study involved a large number of patients and used practical and accurate screening tools, it strengthens the findings of previous studies that reported depression as common in bronchiectasis patients.

A Korean population-based study on bronchiectasis-associated comorbidities reported that the prevalence of depression was 4.3%, but the diagnostic code was used to define comorbidities [6]. Only 4.3% of patients (35/810) in the present study reported a history of depression. These data suggest that comorbidities based on patient self-reports or administrative databases may not reflect the true prevalence of depression. Therefore, we suggest the PHQ-9 for evaluating depression in a baseline study of bronchiectasis.

Differences in depressive symptoms based on bronchiectasis severity were rarely studied. Gao et al. reported that depressive symptoms assessed using HADS did not significantly differ from the degree of severity determined by the BSI and FACED [22]. However, depressive symptoms significantly differed with severity based on the BSI and E-FACED but not FACED in our study. The inconsistency in the results may be attributed to the differences in patient characteristics and depression measurement tools. The E-FACED includes exacerbation, and FACED does not [15, 16]. Our study results showed that exacerbation was a significant factor associated with depression. Therefore, it was hypothesized that the depressive symptoms did not differ with severity based on FACED because there were no items reflecting exacerbation.

The predictive factors for depression in other studies were FEV_1_%, hemoptysis, admission to an emergency department, living with a partner, and sleep disturbance [10, 21, 22]. However, each study included different variables. Some studies collected information on socioeconomic and educational status, but we did not [21, 22]. One study included a separate questionnaire for sleep quality, and questions for sleep disturbance were confined to only one item in the BHQ and PHQ-9 in the present study [22]. Therefore, a comparison between our study and previous studies is not feasible. Dyspnea and a high number of exacerbations were significant predictors of depression in our study. Depression was not related to lung function and obstructive lung diseases. Therefore, bronchiectasis-specific factors may be associated with the deterioration of symptoms and the development of depression. The association between dyspnea and depression seems complex [26]. Depression can affect the perception of dyspnea, and dyspnea can lead to the worsening of depression [27]. Dyspnea is often associated with a decrease in physical activity, which can cause psychological symptoms [26]. However, an association with physical activity was not determined in our study because it was not measured. Depressive symptoms are closely related to frequent exacerbations and poor control of disease in COPD [28]. Similar to COPD, frequent exacerbations of bronchiectasis were associated with poor control of disease. Therefore, the use of inhaled or long-term macrolide antibiotics for the prevention of exacerbation and the management of depression should be considered, as suggested by bronchiectasis guidelines [29].

Patients with chronic diseases showed a higher risk of depression, and patients with depression had poorer clinical outcomes than patients without depression [30, 31]. Although bronchiectasis is the third most common chronic airway disease, the impact of comorbidities was rarely studied [2]. A European observational cohort study suggested the Bronchiectasis Aetiology Comorbidity Index (BACI) [7]. The BACI includes 13 comorbidities that independently predict 5-year mortality. Depression was the tenth most common comorbidity. However, it did not correlate with increased mortality. Some studies reported that depressive symptoms correlated with poor quality-of-life [11, 22]. However, another study did not find a relationship between these two factors [23]. The findings from our study are consistent with a previous finding that depression was closely linked with quality-of-life. Ddepression was strongly correlated with fatigue symptoms. Fatigue is a common symptom in chronic airway disease, including bronchiectasis, and negatively affects exercise tolerance [32]. Physical inactivity is a risk factor for depression [33]. Depression and fatigue were associated with chronic inflammation, which may lead to changes in brain structure or function [34]. Therefore, depression and fatigue commonly manifest simultaneously in chronic inflammatory disease [35]. A meta-analysis showed that fatigue was strongly associated with quality-of-life in bronchiectasis [36]. Therefore, a thorough investigation of patient-reported outcomes for identifying factors affecting quality-of-life is warranted.

The strength of the present study is the large sample size. Most studies included a few patients recruited at a single center [22, 33]. To the best of our knowledge, our study is the first study to demonstrate the prevalence of depression, its clinical relevance, and associated factors based on data obtained from a national multicenter cohort of patients with bronchiectasis. Therefore, our study results are relatively generalizable. This study had several limitations. First, we used different tools to identify depression and included different variables. This difference limits the direct comparison of our results with other studies. Second, although significance of LTOT or NIV was not identified in the multivariate analysis, we could not rule out the significance of that because small proportion of patients treated with LTOT or NIV was included in this study. Third, our study could not explain the causality between depression and bronchiectasis because the results were not based on a prospective study. A prospective study on the impact of depression and the clinical course of bronchiectasis is warranted.

## Conclusion

Depression is common and negatively affects the quality-of-life, but it is underrecognized in bronchiectasis. Active screening for depression is necessary to optimize the treatment of bronchiectasis, especially in patients with risk factors, such as dyspnea and exacerbation.

## Data Availability

Data are available from the KMBARC committee upon reasonable request.

## Abbreviations

COVID-19: Coronavirus disease 2019
BHQ: Bronchiectasis Health Questionnaire
BMI: Body mass index
BSI: Bronchiectasis Severity Index
COPD: Chronic obstructive pulmonary disease
FEV_1_: Forced expiratory volume in the first second
FSS: Fatigue Severity Score
FVC: Forced vital capacity
mMRC: Modified Medical Research Council
NTM: Nontuberculosis mycobacterium
LTOT: Long term oxygen therapy
NIV: Noninvasive ventilation
ICU: Intensive care unit
MV: Mechanical ventilation
PA: *Pseudomonas aeruginosa*
PHQ-9: Patient Health Questionnaire 9

## References

[1] Chalmers JD, Chang AB, Chotirmall SH, Dhar R, McShane PJ (2018). Bronchiectasis. Nat Rev Dis Primers.

[2] Martinez-Garcia MA, Polverino E, Aksamit T (2018). Bronchiectasis and chronic airway disease: it is not just about asthma and COPD. Chest.

[3] Weycker D, Hansen GL, Seifer FD (2017). Prevalence and incidence of noncystic fibrosis bronchiectasis among US adults in 2013. Chron Respir Dis.

[4] Quint JK, Millett ERC, Joshi M, Navaratnam V, Thomas SL, Hurst JR (2016). Changes in the incidence, prevalence and mortality of bronchiectasis in the UK from 2004 to 2013: a population-based cohort study. Eur Respir J.

[5] Lin JL, Xu JF, Qu JM (2016). Bronchiectasis in China. Ann Am Thorac Soc.

[6] Choi H, Yang B, Nam H, Kyoung DS, Sim YS, Park HY (2019). Population-based prevalence of bronchiectasis and associated comorbidities in South Korea. Eur Respir J.

[7] McDonnell MJ, Aliberti S, Goeminne PC, Restrepo MI, Finch S, Pesci A (2016). Comorbidities and the risk of mortality in patients with bronchiectasis: an international multicentre cohort study. Lancet Respir Med.

[8] Su X, Ren Y, Li M, Zhao X, Kong L, Kang J (2016). Prevalence of comorbidities in asthma and nonasthma patients: a meta-analysis. Medicine (Baltimore).

[9] Pelgrim CE, Peterson JD, Gosker HR, Schols A, van Helvoort A, Garssen J (2019). Psychological co-morbidities in COPD: targeting systemic inflammation, a benefit for both?. Eur J Pharmacol.

[10] Ryu YJ, Chun EM, Lee JH, Chang JH (2010). Prevalence of depression and anxiety in outpatients with chronic airway lung disease. Korean J Intern Med.

[11] Olveira C, Olveira G, Gaspar I, Dorado A, Cruz I, Soriguer F (2013). Depression and anxiety symptoms in bronchiectasis: associations with health-related quality of life. Qual Life Res.

[12] Lee H, Choi H, Sim YS, Park S, Kim WJ, Yoo KH (2020). KMBARC registry: protocol for a multicentre observational cohort study on non-cystic fibrosis bronchiectasis in Korea. BMJ Open.

[13] Hill AT, Haworth CS, Aliberti S, Barker A, Blasi F, Boersma W (2017). Pulmonary exacerbation in adults with bronchiectasis: a consensus definition for clinical research. Eur Respir J.

[14] Chalmers JD, Goeminne P, Aliberti S, McDonnell MJ, Lonni S, Davidson J (2014). The bronchiectasis severity index. An international derivation and validation study. Am J Respir Crit Care Med.

[15] Martinez-Garcia MA, de Gracia J, Vendrell Relat M, Giron RM, Maiz Carro L, de la Rosa CD (2014). Multidimensional approach to non-cystic fibrosis bronchiectasis: the FACED score. Eur Respir J.

[16] Martinez-Garcia MA, Athanazio RA, Giron R, Maiz-Carro L, de la Rosa D, Olveira C (2017). Predicting high risk of exacerbations in bronchiectasis: the E-FACED score. Int J Chron Obstruct Pulmon Dis.

[17] Spinou A, Siegert RJ, Guan WJ, Patel AS, Gosker HR, Lee KK (2017). The development and validation of the Bronchiectasis Health Questionnaire. Eur Respir J.

[18] Kim HK, Lee H, Kim SH, Choi H, Lee JH, Lee JS (2020). Validation of the Korean version of the bronchiectasis health questionnaire. Tuberc Respir Dis (Seoul).

[19] Lee JH, Jeong HS, Lim SM, Cho HB, Ma JY, Ko E (2013). Reliability and validity of the fatigue severity scale among university student in South Korea. Korean J Biol Psychiatry.

[20] Park SJ, Choi HR, Choi JH, Kim KW, Hong JP (2010). Reliability and validity of the Korean version of the Patient Health Questionnaire-9 (PHQ-9). Anxiety and Mood.

[21] Ozgun Niksarlioglu EY, Ozkan G, Gunluoglu G, Uysal MA, Gul S, Kilic L (2016). Factors related to depression and anxiety in adults with bronchiectasis. Neuropsychiatr Dis Treat.

[22] Gao YH, Guan WJ, Zhu YN, Chen RC, Zhang GJ (2018). Anxiety and depression in adult outpatients with bronchiectasis: associations with disease severity and health-related quality of life. Clin Respir J.

[23] Girón Moreno RM, Fernandes Vasconcelos G, Cisneros C, Gómez-Punter RM, Segrelles Calvo G, Ancochea J (2013). Presence of anxiety and depression in patients with bronchiectasis unrelated to cystic fibrosis. Arch Bronconeumol (Engl Ed).

[24] Nowak C, Sievi NA, Clarenbach CF, Schwarz EI, Schlatzer C, Brack T (2014). Accuracy of the Hospital Anxiety and Depression Scale for identifying depression in chronic obstructive pulmonary disease patients. Pulm Med.

[25] Levis B, Benedetti A, Thombs BD, Collaboration DESD (2019). Accuracy of Patient Health Questionnaire-9 (PHQ-9) for screening to detect major depression: individual participant data meta-analysis. BMJ.

[26] Schuler M, Wittmann M, Faller H, Schultz K (2018). The interrelations among aspects of dyspnea and symptoms of depression in COPD patients—a network analysis. J Affect Disord.

[27] Borges-Santos E, Wada JT, da Silva CM, Silva RA, Stelmach R, Carvalho CR (2015). Anxiety and depression are related to dyspnea and clinical control but not with thoracoabdominal mechanics in patients with COPD. Respir Physiol Neurobiol.

[28] Pooler A, Beech R (2014). Examining the relationship between anxiety and depression and exacerbations of COPD which result in hospital admission: a systematic review. Int J Chron Obstruct Pulmon Dis.

[29] Hill AT, Sullivan AL, Chalmers JD, De Soyza A, Elborn JS, Floto RA (2018). British Thoracic Society guideline for bronchiectasis in adults. BMJ Open Respir Res.

[30] Moussavi S, Chatterji S, Verdes E, Tandon A, Patel V, Ustun B (2007). Depression, chronic diseases, and decrements in health: results from the World Health Surveys. Lancet.

[31] Divo M, Celli BR (2020). Multimorbidity in patients with chronic obstructive pulmonary disease. Clin Chest Med.

[32] Ozalp O, Inal-Ince D, Calik E, Vardar-Yagli N, Saglam M, Savci S (2012). Extrapulmonary features of bronchiectasis: muscle function, exercise capacity, fatigue, and health status. Multidiscip Respir Med.

[33] Kim SY, Park JH, Lee MY, Oh KS, Shin DW, Shin YC (2019). Physical activity and the prevention of depression: a cohort study. Gen Hosp Psychiatry.

[34] Lee CH, Giuliani F (2019). The role of inflammation in depression and fatigue. Front Immunol.

[35] Corfield EC, Martin NG, Nyholt DR (2016). Co-occurrence and symptomatology of fatigue and depression. Compr Psychiatry.

[36] Spinou A, Fragkos KC, Lee KK, Elston C, Siegert RJ, Loebinger MR (2016). The validity of health-related quality of life questionnaires in bronchiectasis: a systematic review and meta-analysis. Thorax.

